# Supplementary material for: Astrochronology of the Paleocene-Eocene Thermal Maximum on the Atlantic Coastal Plain
Source: Nat Commun. 2022 Sep 24;13:5618. doi: 10.1038/s41467-022-33390-x (PMC9509358; doi:10.1038/s41467-022-33390-x)
Supplement: Supplementary file 1 — Supplementary Information [file 41467_2022_33390_MOESM1_ESM.pdf]

**Supplementary Information for**

# Astrochronology of the Paleocene-Eocene Thermal Maximum on the Atlantic Coastal Plain

Mingsong Li*, Timothy J. Bralower, Lee R. Kump, Jean M. Self-Trail, James C. Zachos, William D. Rush, Marci M. Robinson

* Correspondence and requests for materials should be addressed to M.L. (email: msli@pku.edu.cn)

The Supplementary Information includes:

- Supplementary Figures 1-18
- Supplementary Table 1
- Supplementary Text 1 Changepoint analysis
- Supplementary Text 2 Settings and outputs of the average spectral misfit analysis
- Supplementary Text 3 Correlation coefficient (COCO) analysis
- Supplementary References

## Supplementary Figures

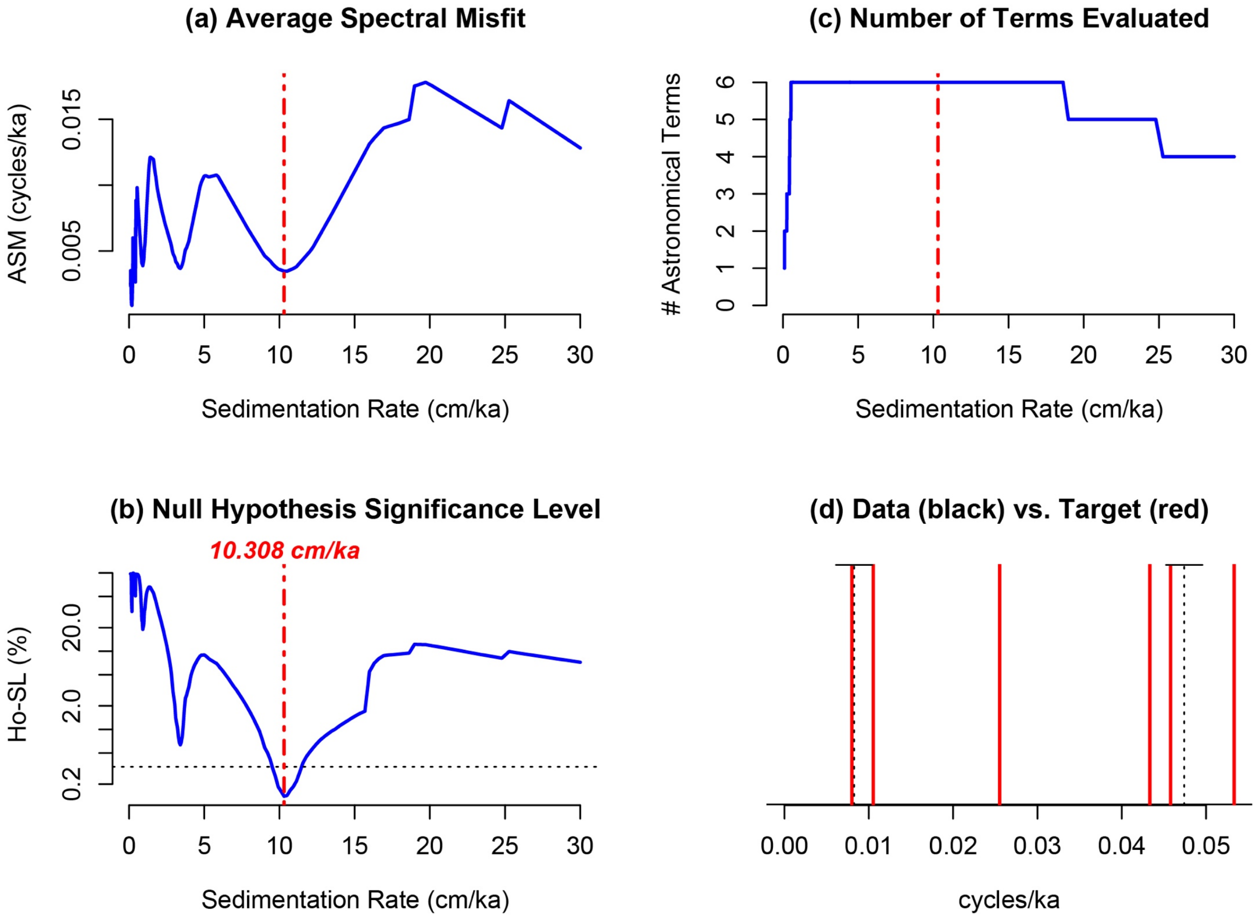


**Supplementary Figure 1** Average spectral misfit (ASM) of the detrended $\log_{10}$(Ca) at Howards Tract cores. The series is interpolated to a 0.05 cm sampling rate after removing a linear trend. The parameters are detailed in Supplementary Text 1.

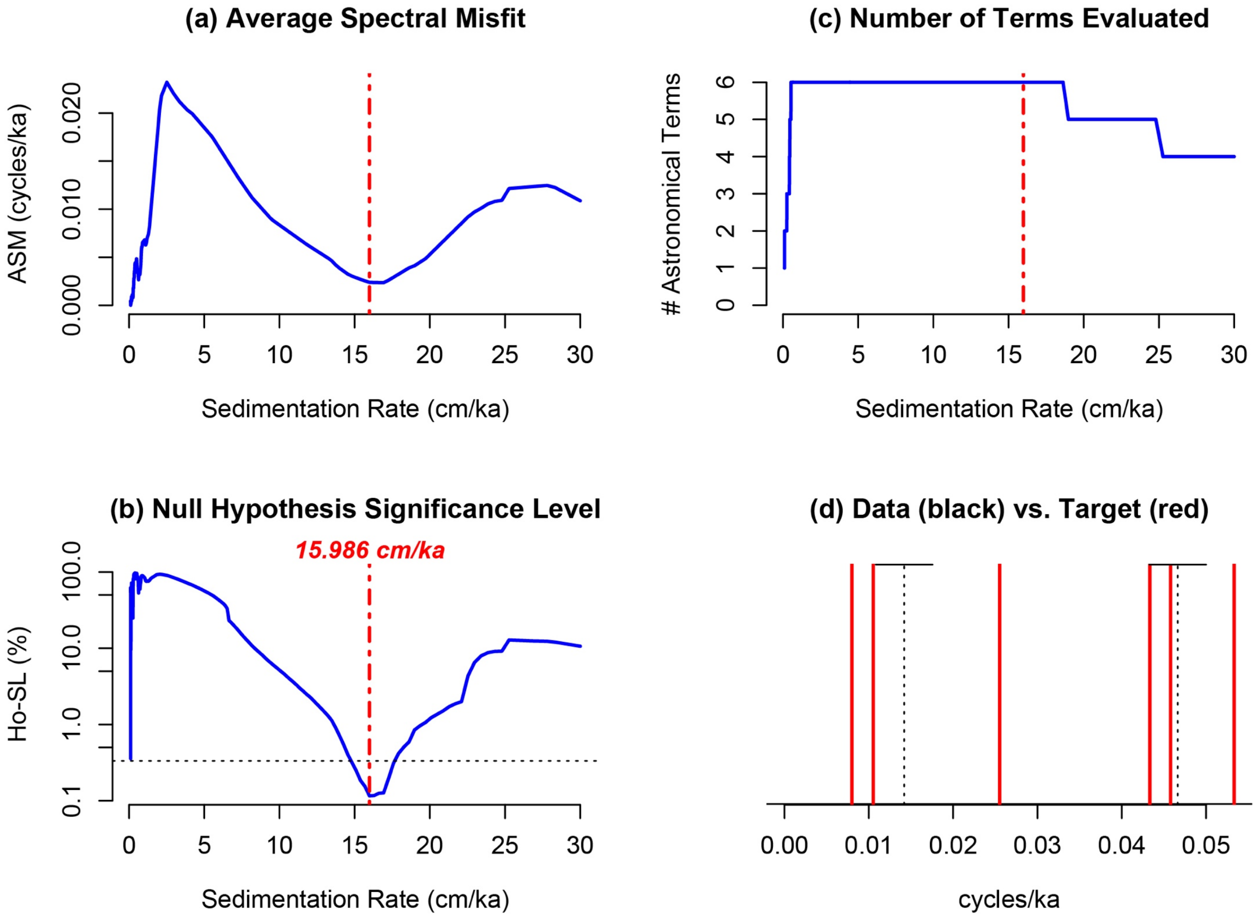


**Supplementary Figure 2** Average spectral misfit (ASM) of the magnetic susceptibility at Howards Tract cores. The series is interpolated to a 0.05 cm sampling rate after removing a 20 m loess trend. The parameters are detailed in Supplementary Text 1.

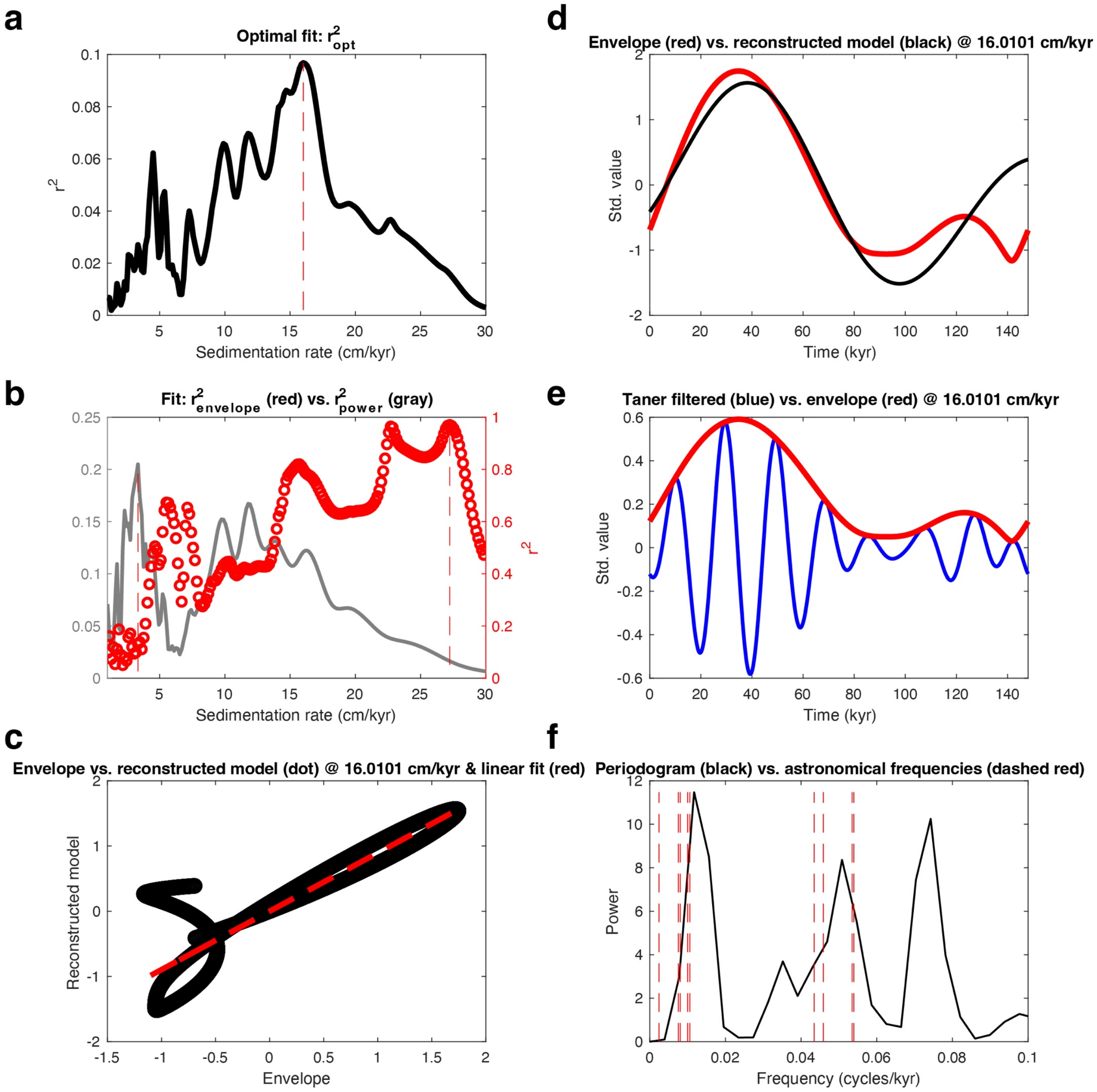


**Supplementary Figure 3** TimeOpt analysis of the linear-detrended $\log_{10}$(Ca) series of the Aquia Formation and the Marlboro Clay. **a** Combined envelope and spectral power fit ($r^2_{opt}$) indicates the optimal sedimentation rate is at 16.0 cm/kyr. **b** Squared correlation coefficient for the amplitude envelope fit ($r^2_{envelope}$) and the spectral power fit ($r^2_{power}$) at test sedimentation rates. **c** Linear fit of amplitude envelope in **e** and the reconstructed eccentricity model in **d**. **d** Comparison of the envelope of the amplitude modulation in **e** and the reconstructed eccentricity model. **e** Filtered precession cycles and their amplitude modulation of the tuned proxy series using a sedimentation rate of 16.0 cm/kyr. **f** Periodogram model of the tuned proxy series using a sedimentation rate of 16.0 cm/kyr shown with specified precession and eccentricity frequencies.

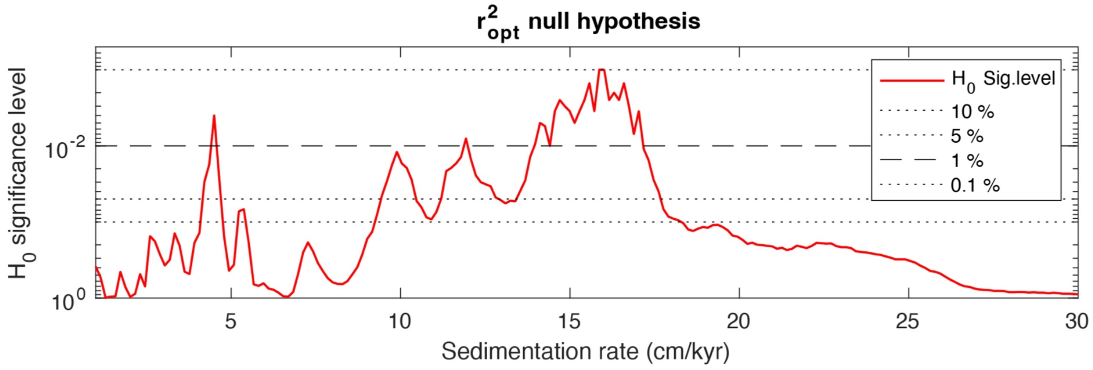


**Supplementary Figure 4** Null hypothesis significance levels of test sedimentation rates for Ca series of the Aquia Formation and the Marlboro Clay. Test sedimentation rates range from 1 cm/kyr to 30 cm/kyr. The total number of test sedimentation rates is 100. The target age for the astronomical frequencies is 55 Ma. Monte Carlo simulation is 2,000.

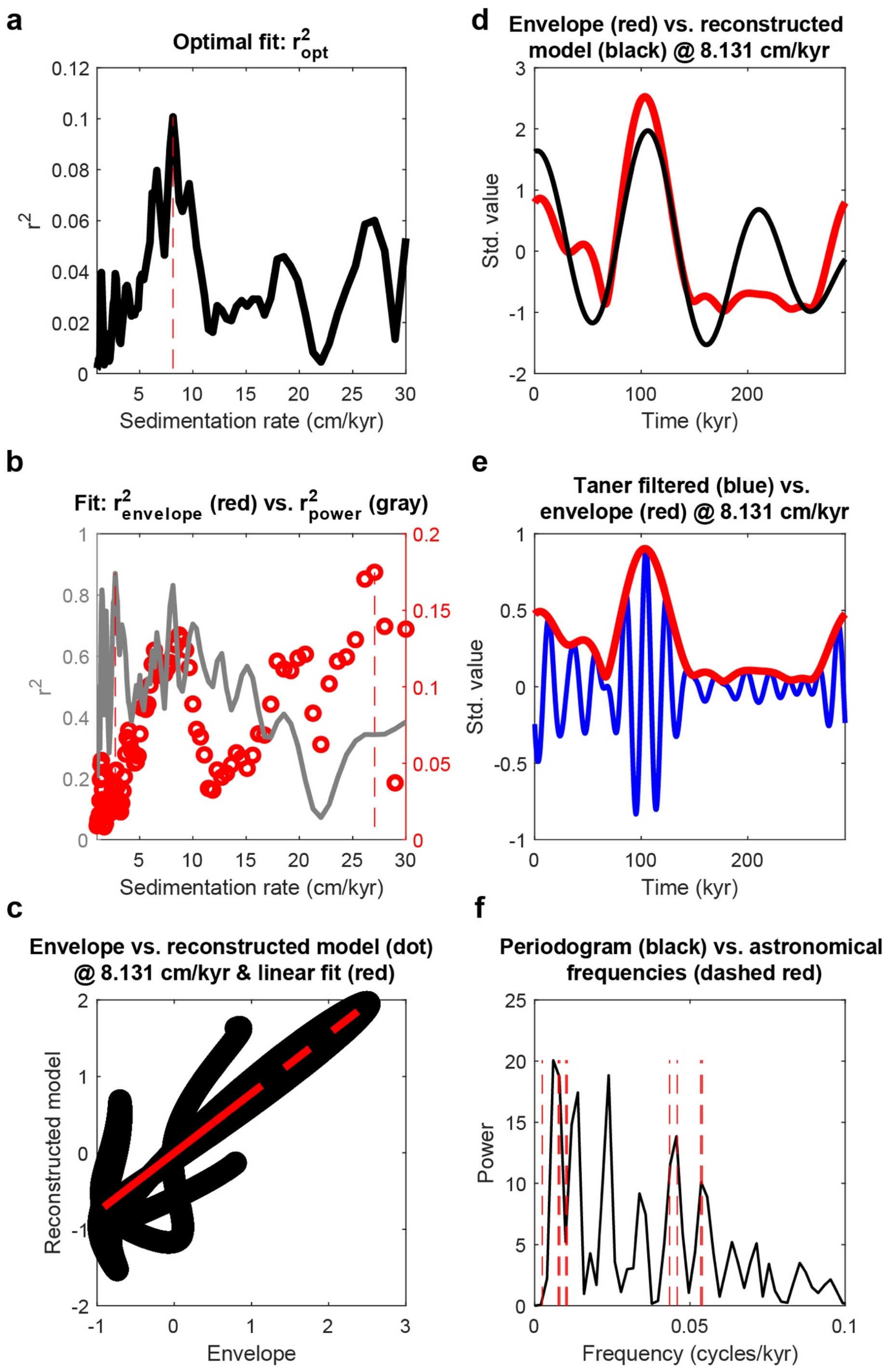


**Supplementary Figure 5** TimeOpt analysis of the magnetic susceptibility (MS) series of the Aquia Formation and the Marlboro Clay. **a** Combined envelope and spectral power fit ($r^2_{opt}$) indicates the optimal sedimentation rate is at 8.13 cm/kyr. **b** Squared correlation coefficient for the amplitude envelope fit ($r^2_{envelope}$) and the spectral power fit ($r^2_{power}$) at test sedimentation rates. **c** Linear fit of amplitude envelope in **e** and the reconstructed eccentricity model in **d**. **d** Comparison of the envelope of the amplitude modulation in **e** and the reconstructed eccentricity model. **e** Filtered precession cycles and their amplitude modulation of the tuned proxy series using a sedimentation rate of 8.13 cm/kyr. **f** Periodogram model of the tuned proxy series using a sedimentation rate of 8.13 cm/kyr shown with specified precession and eccentricity frequencies.

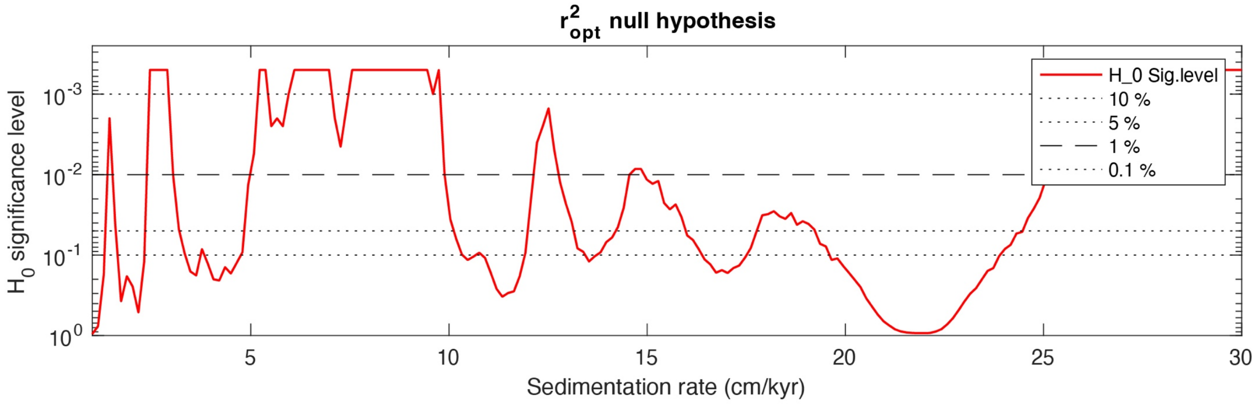


**Supplementary Figure 6** Null hypothesis significance levels of test sedimentation rates for magnetic susceptibility (MS) series of the Aquia Formation and the Marlboro Clay. Test sedimentation rates range from 1 cm/kyr to 30 cm/kyr. The total number of test sedimentation rates is 100. The target age for the astronomical frequencies is 55 Ma. Monte Carlo simulation is 2,000.

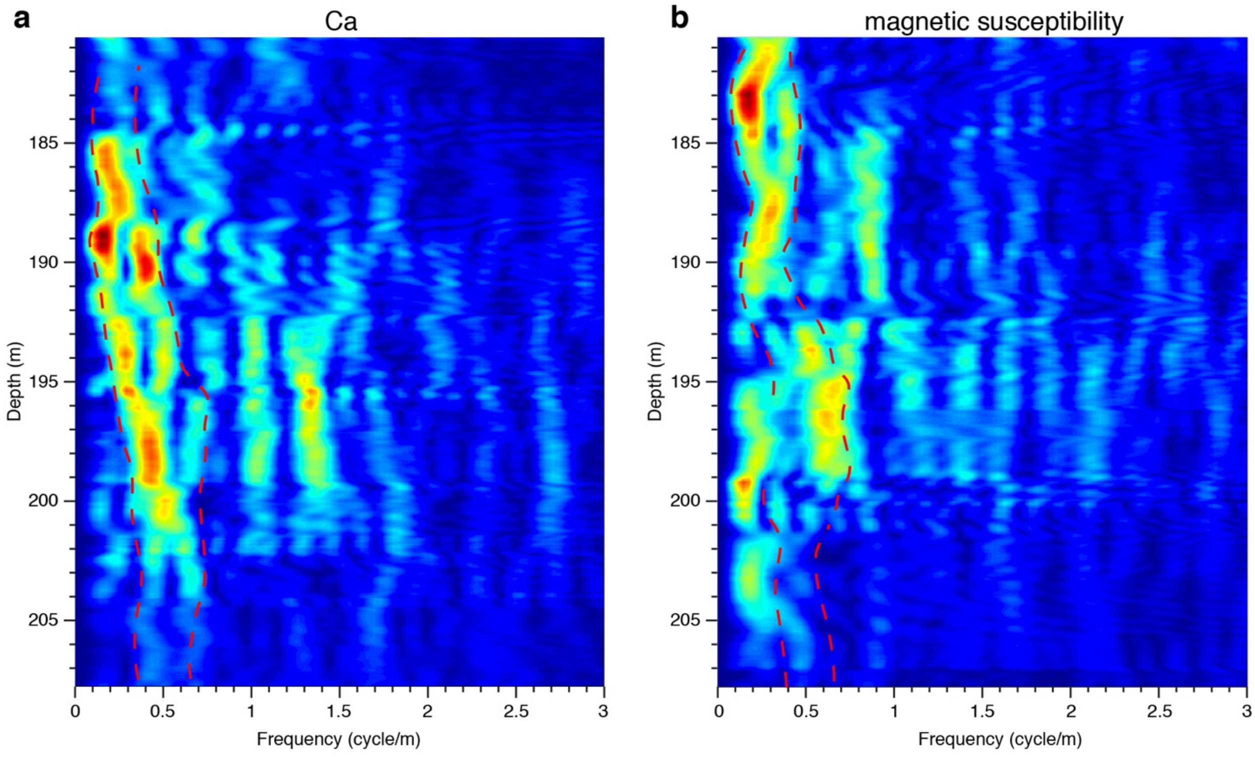


**Supplementary Figure 7** Evolutionary fast Fourier transform (FFT) of the detrended $\log_{10}$(Ca) content (**a**) and detrended magnetic susceptibility (**b**) at Howards Tract. Dashed red lines bound signals of precession cycles. Both series are interpolated to a 0.005 cm sampling rate. Sliding window is 7 m with a step of 0.01 m.

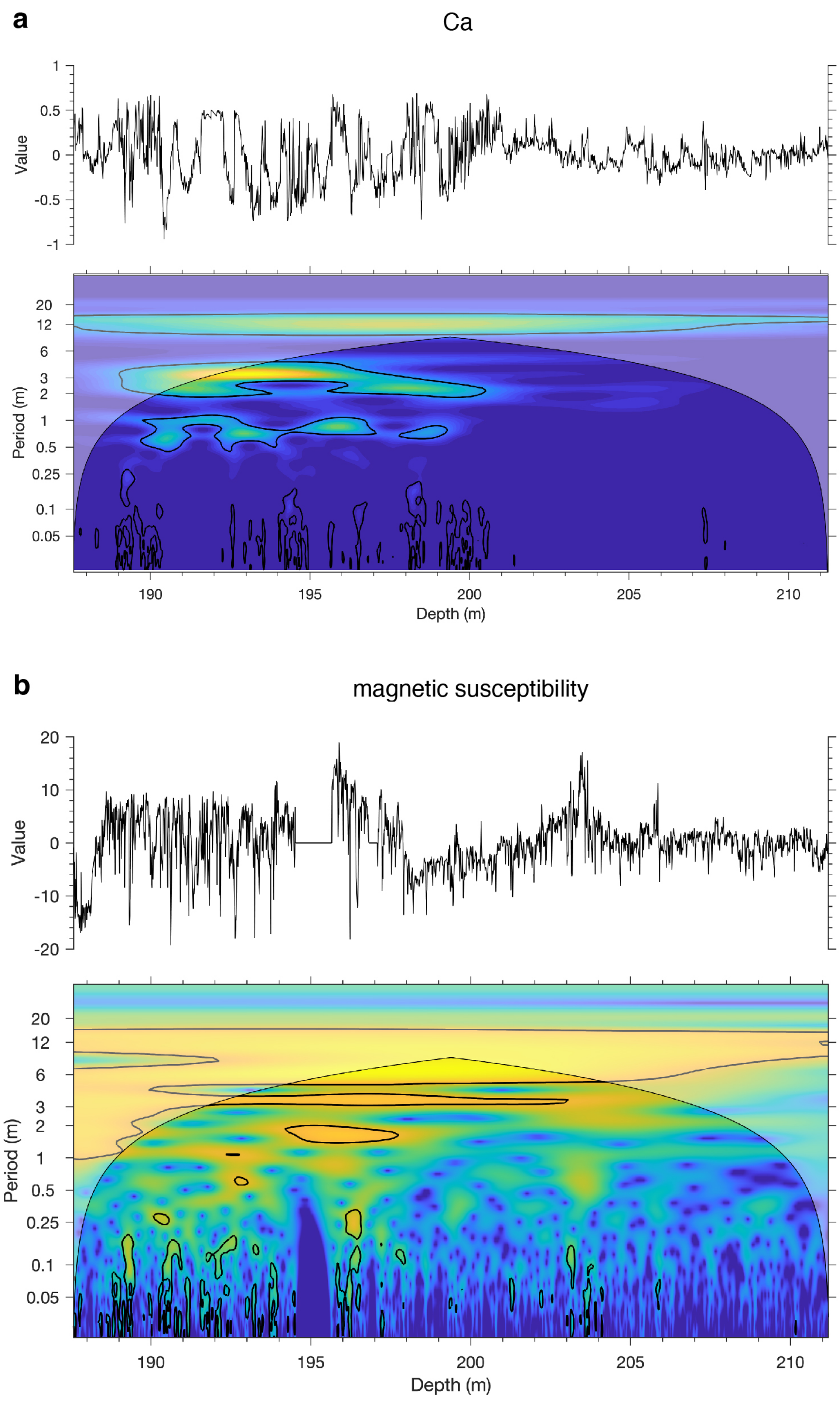


**Supplementary Figure 8** Wavelet transform of the detrended $\log_{10}$(Ca) content (**a**) and detrended magnetic susceptibility (**b**) at Howards Tract using Acycle v2.4.1 and scripts provided in ref. [1]. Black lines denote significance level of 0.05 and shaded region denotes cone of influence.

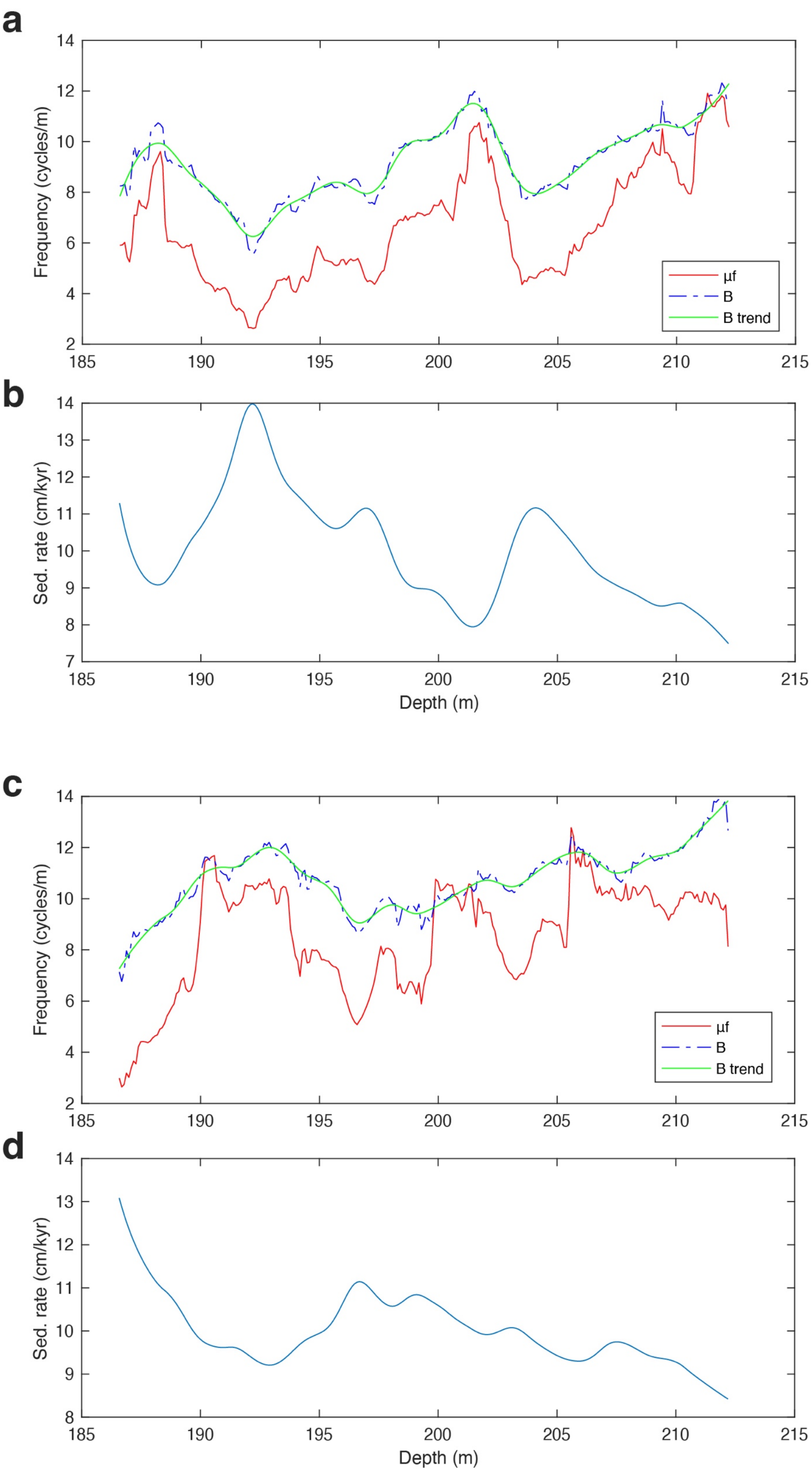


**Supplementary Figure 9** Spectral moments of $\log_{10}$(Ca) (**a-b**) and magnetic susceptibility (**c-d**) series. **a** mean frequency (μf) and bandwidth (B) of the $\log_{10}$(Ca) series shown with the 4-m LOESS trend of the bandwidth (B trend). **b** Scaled sedimentation rate for the $\log_{10}$(Ca) series. **c** mean frequency (μf) and bandwidth (B) of the magnetic susceptibility series shown with the 4 -m LOESS trend of the bandwidth (B trend). **d** Scaled sedimentation rate for the magnetic susceptibility series.

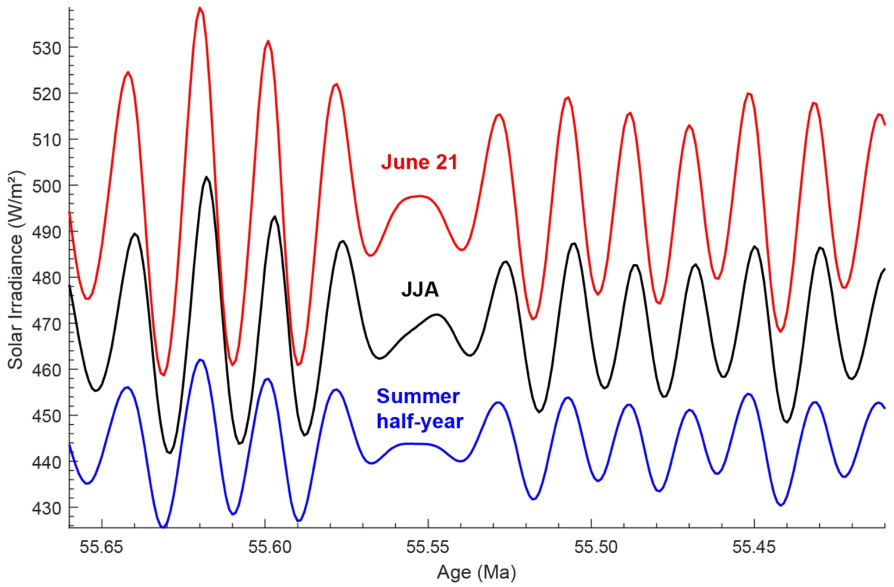


**Supplementary Figure 10** Mean solar irradiance at 35.685° N, which is the latitude of the grid including Howards Tract cores in cGENIE model on June 21 (red), in June-July-August (black), and in summer half-year (blue). All these curves are paced by ~20 kyr precession cycles modulated by eccentricity cycles. The irradiance data were calculated using the "Insolation" function in Acycle software.

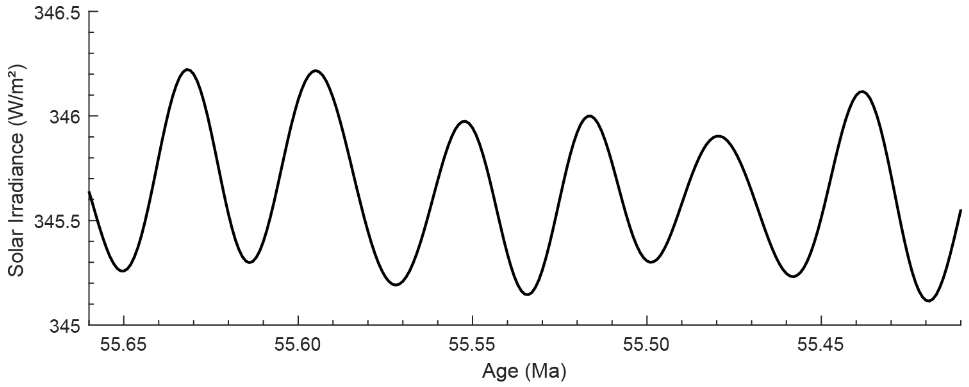


**Supplementary Figure 11** Annual solar irradiance at 35.685° N, which is the latitude of the grid including Howards Tract cores in cGENIE model. This curve is paced by 40 kyr obliquity cycles. The irradiance data were calculated using the "Insolation" function in Acycle software.

HT2

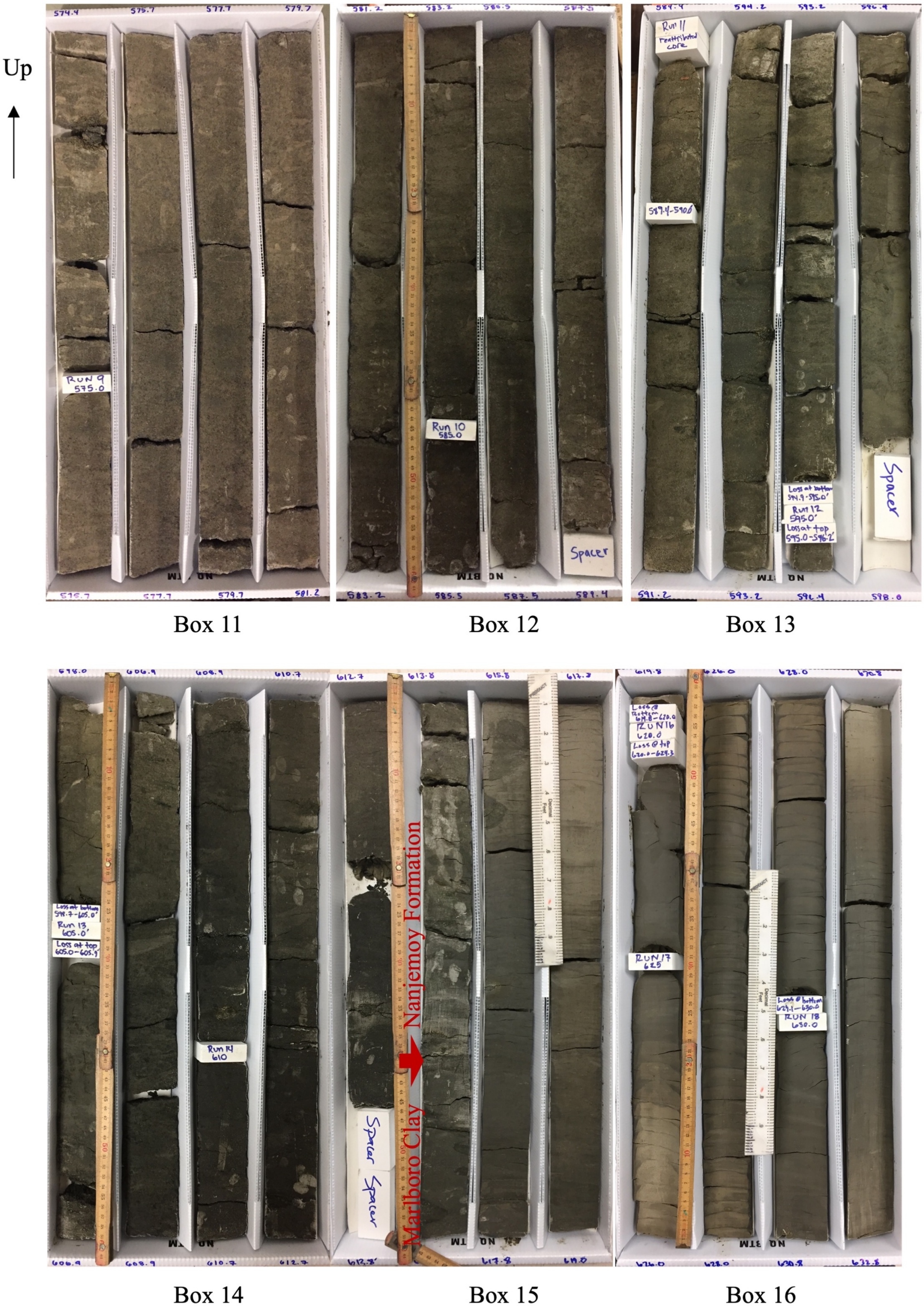


**Supplementary Figure 12** Core photos. Howards Tract 2 (HT2) core from 175.08 m (574.4 ft) to 192.88 m (632.8 ft).

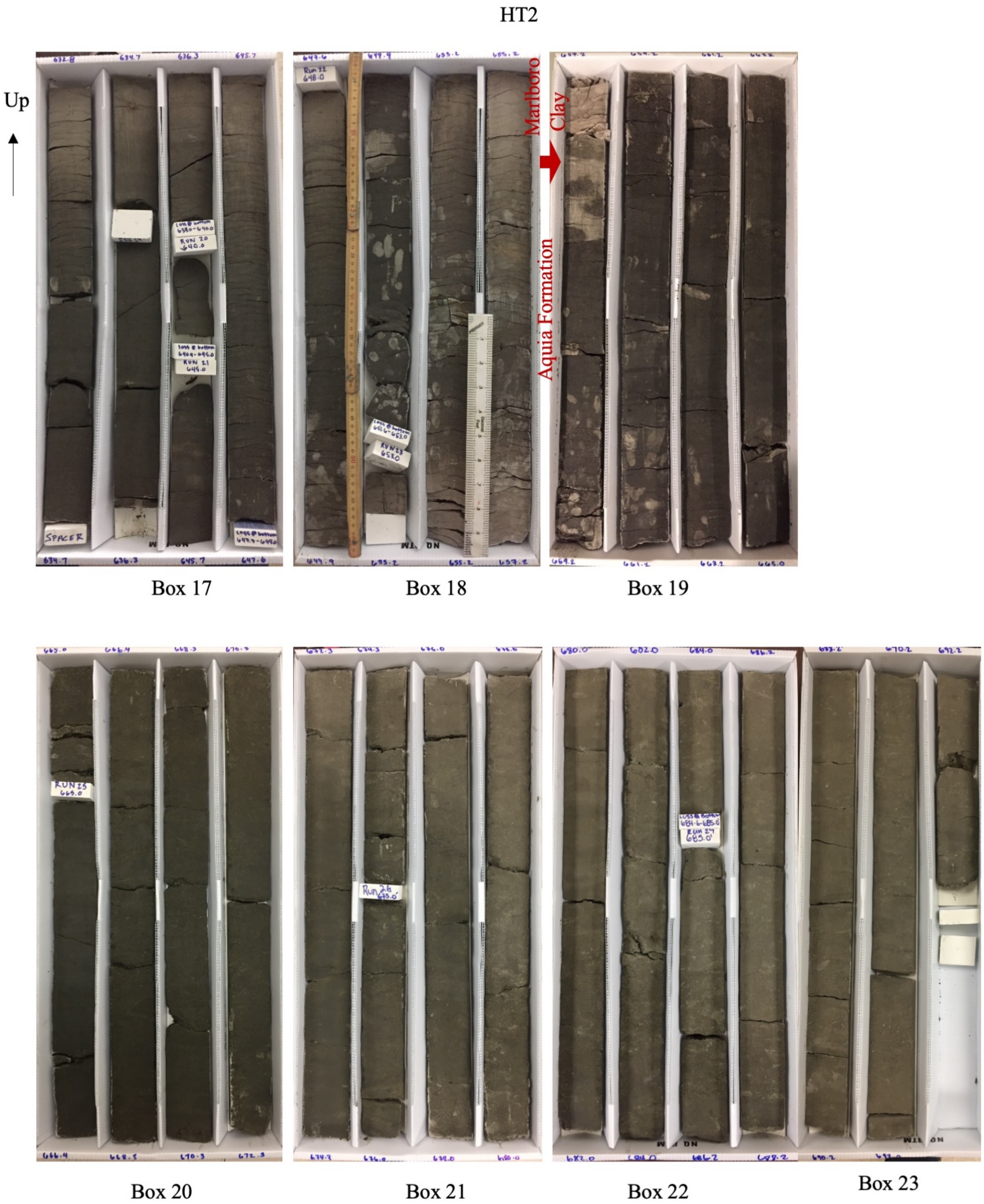


**Supplementary Figure 13** Core photos. Howards Tract 2 (HT2) core from 192.88 m (632.8 ft) to 211.24 m (693.0 ft). The light gray dots and areas in boxes 18 and 19 don't reflect original color but the color after the muddy water has dried.

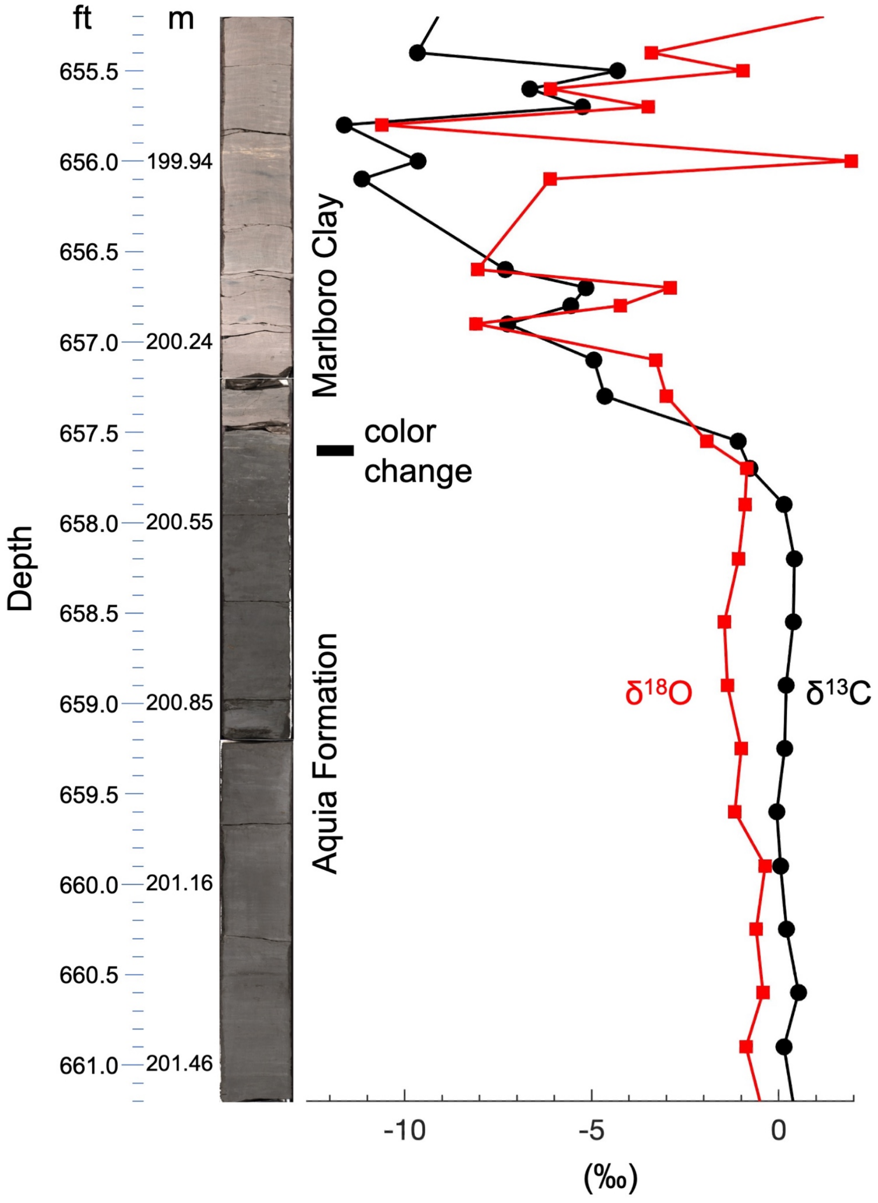


**Supplementary Figure 14** High-resolution core photos near the Aquia/Marlboro contact shown with $\delta^{13}C$ (black dot) and $\delta^{18}O$ (red square) data. The sediments change from a silty clay above (Dark greenish gray 5g4/1; Marlboro Clay) to a glauconitic sandy clay below (Greenish black 5gy 2/1; Aquia Formation) with no obvious break. Additionally, there isn't a big changeover in the nannoflora that would suggest missing time.

HT1

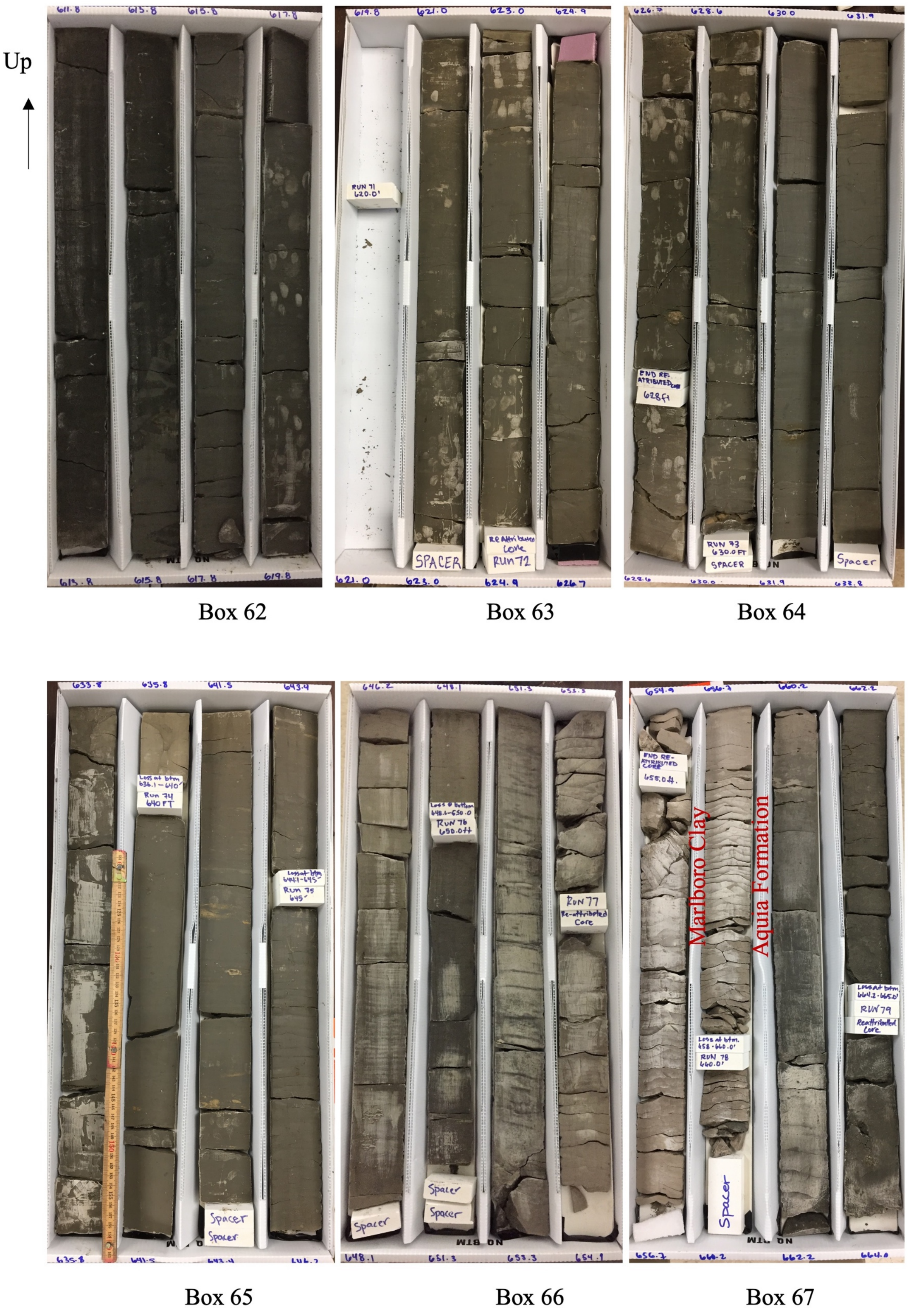


**Supplementary Figure 15** Core photos. Howards Tract 1 (HT1) core from 186.48 m (611.8 ft) to 202.39 m (664.0 ft).

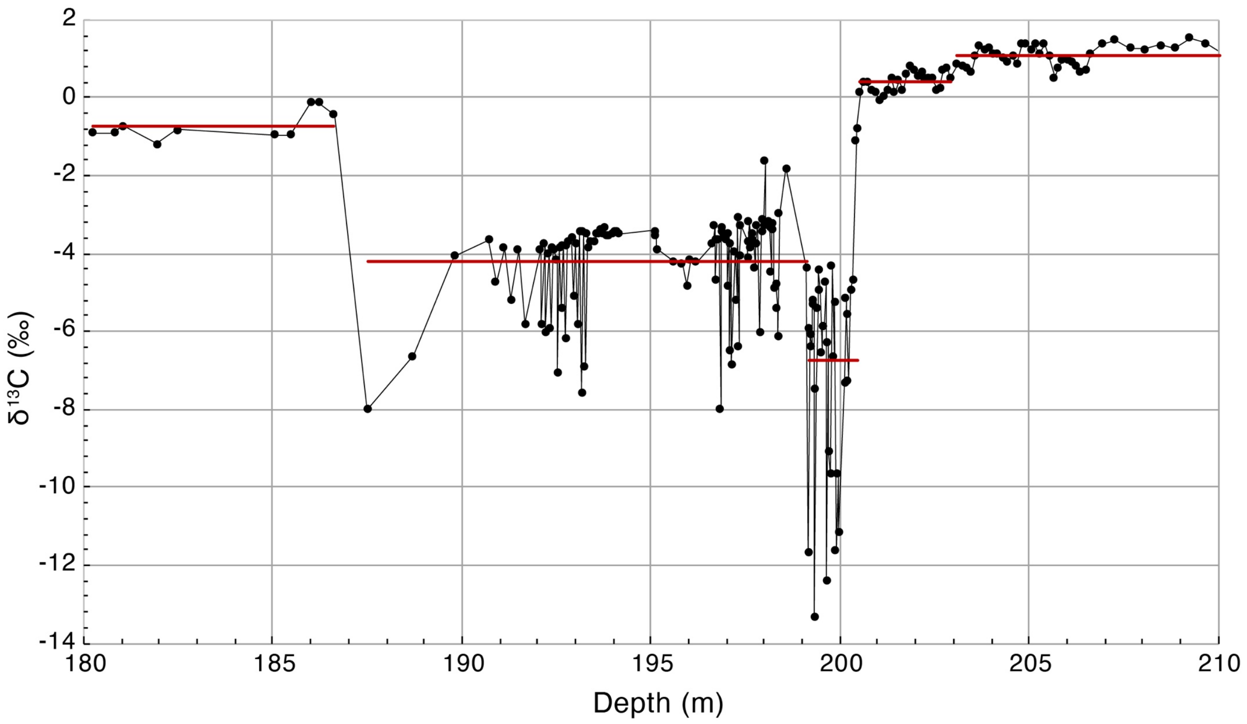


**Supplementary Figure 16** Change point analysis of the $\delta^{13}C$ data. See Supplementary Text 1 for details.

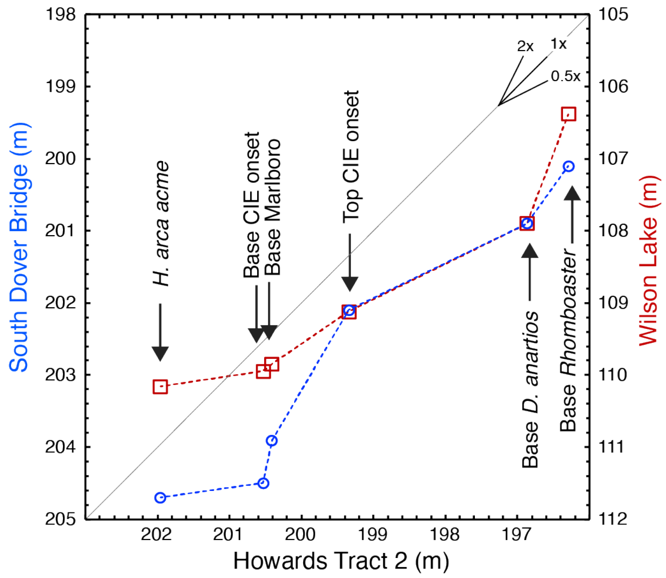


**Supplementary Figure 17** Comparison of the carbon isotope excursion (CIE) onset with the base of the Marlboro Clay as well as three nannoplankton datums. Among these, the top of the CIE onset uses a less likely option (199.34 m). Blue: Howards Tract 2 versus South Dover Bridge (left); Red: Howards Tract 2 versus Wilson Lake (right).

The analysis of the timing of the CIE onset relative to biostratigraphic datums and the base of the Marlboro Clay at Howards Tract 2 (HT2), South Dover Bridge (SDB), Maryland and Wilson Lake (WL), New Jersey is shown in Figure 5 and Supplementary Figure 17. The biostratigraphic datums are from ref. [2] for SDB, ref. [3] and ref. [4] for WL and Self-Trail (unpublished data) for HT2. The base of the Marlboro Clay is based on ref. [5]. The analysis used here is based on ref. [6].

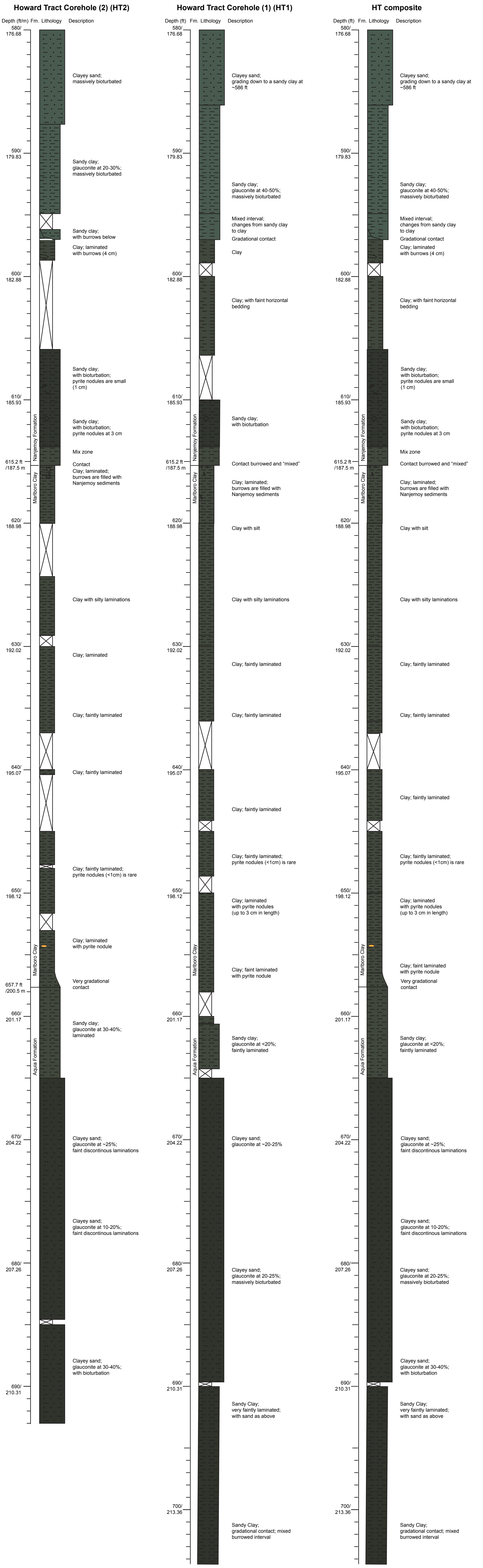


**Supplementary Figure 18** Lithology of Howards Tract cores.

**Supplementary Table 1** Age model for the Howards Tract cores. ATS: astronomical time scale.

| **Depth (m)** | **Time (kyr, floating ATS)** | **Label (precession no.)** | **Sedimentation rate (cm/kyr)** |
|---|---|---|---|
| 186.707 | 170.156 | P6 | 15.6 |
| 189.572 | 151.84 | P5 | 11.1 |
| 192.037 | 129.668 | P4 | 7.8 |
| 195.872 | 80.263 | P2 | 12.1 |
| 198.417 | 59.296 | P1 | 11.1 |
| 200.662 | 39.052 | P0 | 8.3 |
| 202.592 | 15.675 | P-1 | 11.6 |
| 205.102 | -6 | P-2 | 9.5 |
| 207.392 | -30 | P-3 | 15.9 |
| 210.727 | -51 | P-4 | |

**Supplementary Text 1 Changepoint analysis**

Four changepoints are detected at locations of 186.61, 199.12, 200.47, and 202.92 m (Supplementary Fig. 16) using the settings and codes below.

```
> filename = "/to/HT-d13C.txt"
> x = read.delim(filename, header = FALSE, sep = ",", dec =
".")[ ,2]
> ans =
cpt.meanvar(x,penalty="Manual",pen.value="10*log(n)",method="P
ELT")
> plot(ans,cpt.width=2)
> print(ans)
Class 'cpt' : Changepoint Object
       ~~   : S4 class containing 12 slots with names
              cpttype date version data.set method test.stat
pen.type pen.value minseglen cpts ncpts.max param.est

Created on  : Wed Jul 15 03:20:23 2020

summary(.)  :
----------
Created Using changepoint version 2.2.2
Changepoint type      : Change in mean and variance
Method of analysis    : PELT
Test Statistic  : Normal
Type of penalty       : Manual with value, 53.93628
Minimum Segment Length : 2
Maximum no. of cpts   : Inf
Changepoint Locations : 10 116 149 174

# Changepoint Locations (m): 186.61 199.12 200.47 202.92
```

**Supplementary Text 2 Settings and outputs of the average spectral misfit analysis**

Average spectral misfit (ASM) results are shown in **Fig. 4** in the main text and **Supplementary Figures 1** and **2**. Detailed settings for the ASM analysis are listed below.

**Calcium content**

```
> data<-read()   # select HT-Ca-su-187.54+-log10-Linear.txt
> dat = linterp(data,dt = .05)
>
freq=lowspec(dat,detrend=T,padfac=10,siglevel=0.9,output=2,xma
x=10)

* Number of significant F-test peaks identified = 6
ID  / Frequency / Period / Harmonic_CL / LOWSPEC_CL
1   0.08016878   12.47368   99.8097   58.81714
2   0.4599156   2.174312   99.45369   64.70208
3   1.362869   0.7337461   92.89494   99.14315
4   1.49789   0.6676056   97.05687   96.20943
```

```
5   5.042194   0.1983264   97.82874   91.03616
6   5.413502   0.1847233   90.23107   94.15875
----- PERFORMING Robust Locally-Weighted Regression Spectral
Background Estimation -----
   * Number of data points in stratigraphic series: 475
   * Stratigraphic series length (space or time): 23.7
   * Sampling interval (space or time): 0.05

   * Raw AR1 = 0.6855143
   * Prewhitening with AR1 coefficient of 0.6855143
   * Prewhitened AR1 = -0.09220875

   * Linear trend removed. m= -8.854628e-05 b= 0.01900229

   * Nyquist frequency: 10
   * Rayleigh frequency: 0.04219409
   *  MTM  Power  spectrum  bandwidth  resolution  (halfwidth):
0.1265823
   * Padded to 4740 points
   * LOWSPEC tuning parameter = 5.360768

   * Searching for significant spectral peaks that satisfy 90 %
CL
      requirements outlined in Meyers (2012):
   * Number of significant F-test peaks identified = 6
  ID  / Frequency / Period / Harmonic_CL / LOWSPEC_CL
  1   0.08016878   12.47368   99.8097   58.81714
  2   0.4599156   2.174312   99.45369   64.70208
  3   1.362869   0.7337461   92.89494   99.14315
  4   1.49789   0.6676056   97.05687   96.20943
  5   5.042194   0.1983264   97.82874   91.03616
  6   5.413502   0.1847233   90.23107   94.15875

  >                                                       freqs
=c(0.08016878,0.4599156,1.362869,1.49789,5.042194,5.413502)
  > target= c(1/125, 1/95, 1/39.17,1/23.08,1/21.84, 1/18.75)
  >   asm(freq=freqs,   target=target,   rayleigh=   0.04219409,
nyquist= 10,sedmin = 0.1,sedmax = 30, numsed = 300, iter=100000)

  ----- PERFORMING AVERAGE SPECTRAL MISFIT ANALYSIS -----

  **** WARNING: No uncertainty assigned to astronomical target
frequencies.

   * Analysis complete:
      Optimal Sedimentation Rate (cm/ka) at = 10.3081
      Ho-SL (%) = 0.14
        or p-value = 0.0014
      ASM (cycles/ka) = 0.003472542
      Number of Astronomical Terms Fit = 6
```

**Magnetic susceptibility**

```
> data<-read()  # read HT-MS-comp-187.54-211.24-20-LOESS.txt
> dat = linterp(data,dt = .05)

----- APPLYING PIECEWISE-LINEAR INTERPOLATION TO STRATIGRAPHIC
SERIES -----

 * Number of samples= 4364
 * New number of samples= 474
>
freq=lowspec(dat,detrend=T,padfac=10,siglevel=0.9,output=2,xma
x=10)

----- PERFORMING Robust Locally-Weighted Regression Spectral
Background Estimation -----
 * Number of data points in stratigraphic series: 474
 * Stratigraphic series length (space or time): 23.65
 * Sampling interval (space or time): 0.05

 * Raw AR1 = 0.5107695
 * Prewhitening with AR1 coefficient of 0.5107695
 * Prewhitened AR1 = -0.1560995

 * Linear trend removed. m= 0.02044113 b= -4.145343

 * Nyquist frequency: 10
 * Rayleigh frequency: 0.0422833
 * MTM Power spectrum bandwidth resolution (halfwidth):
0.1268499
 * Padded to 4730 points
 * LOWSPEC tuning parameter = 5.355136

 * Searching for significant spectral peaks that satisfy 90 %
CL
     requirements outlined in Meyers (2012):
 * Number of significant F-test peaks identified = 10
ID  / Frequency / Period / Harmonic_CL / LOWSPEC_CL
1   0.08879493   11.2619   93.48088   99.96105
2   0.2917548   3.427536   99.92273   63.46254
3   3.94926   0.253212   94.45833   81.81575
4   4.097252   0.244066   91.24796   65.77968
5   6.118393   0.1634416   94.7472   94.0295
6   6.824524   0.1465304   90.99411   86.75781
7   7.391121   0.1352975   98.83166   94.73402
8   8.372093   0.1194444   90.98938   98.2276
9   9.72093   0.1028708   90.63362   92.49079
10   9.915433   0.1008529   99.26139   94.63583
> freqs = c(0.08879493, 0.2917548, 3.94926, 4.097252, 6.118393,
6.824524, 7.391121, 8.372093, 9.72093, 9.915433)
> target= c(1/125, 1/95, 1/39.17,1/23.08,1/21.84, 1/18.75)
> asm(freq=freqs, target=target, rayleigh= 0.0422833, nyquist=
10,sedmin = 0.1,sedmax = 30, numsed = 300, iter=100000)
```

```
----- PERFORMING AVERAGE SPECTRAL MISFIT ANALYSIS -----

**** WARNING: No uncertainty assigned to astronomical target
frequencies.

 * Analysis complete:
    Optimal Sedimentation Rate (cm/ka) at = 15.98551
    Ho-SL (%) = 0.116
      or p-value = 0.00116
    ASM (cycles/ka) = 0.002395696
  Number of Astronomical Terms Fit = 6
```

**Supplementary Text 3 Correlation coefficient (COCO) analysis**

Correlation coefficients (COCO) between the power spectrum of the detrended stratigraphic series and the target astronomical solutions are calculated for the test sedimentation rate. The log file for the COCO analyses of Ca and magnetic susceptibility series records the parameters used in the analyses. Results of the COCO analyses are shown in **Fig. 4** in the main paper.

**Calcium content**

```
- - - - - - - - - - - - - - Summary - - - - - - - - - - - -
24-Dec-2021 10:41:02
HT-Ca-su-187.54+-log10-Linear-rsp0.01-linear-2000sim-1slice-
COCO-log.txt
Data: 187.5405 to 211.2405m.
Sampling rate: 0.01.
Number of data points: 2371
Data: Number of slices is 1.
Number of simulations is 2000
Data: Remove red noise model: classic AR1 removed (F - Fred).
Correlation method: Pearson
Zero padding for the data is 5000
Tested sedimentation rate step is 0.1 cm/kyr from 0.13 to 30
cm/kyr
Target age is 56000 ka.
Zero padding is 5000.
Freq. is 0-0.06 cycles/kyr.
Astronomical solution: User-defined
Astronomical cycles are: 125 95 39.8 23.3 22 18.7
 - - - - - - - - - - - - - - - End - - - - - - - - - - - - -
```

**Magnetic susceptibility**

```
- - - - - - - - - - - - - - Summary - - - - - - - - - - - -
24-Dec-2021 11:09:31
HT-MS-comp-187.54-211.24-20-LOESS-rsp0.01-linear-2000sim-
1slice-COCO-log.txt
Data: 187.542 to 211.232m.
Sampling rate: 0.01.
Number of data points: 2370
```

```
Data: Number of slices is 1.
Number of simulations is 2000.
Data: Remove red noise model: classic AR1 removed (F - Fred).
Correlation method: Pearson.
Zero padding for the data is 5000.
Tested sedimentation rate step is 0.1 cm/kyr from 0.13 to 30
cm/kyr.
Target age is 56000 ka.
Zero padding is 5000.
Freq. is 0-0.06 cycles/kyr.
Astronomical solution: User-defined
Astronomical cycles are: 125 95 39.8 23.3 22 18.7
 - - - - - - - - - - - - - - - End - - - - - - - - - - - - -
```

**Supplementary References**


1. Torrence C. & Compo G. P. A practical guide to wavelet analysis. *Bull. Amer. Meteor. Soc.* **79**, 61-78 (1998).
2. Self-Trail J. M., Powars D. S., Watkins D. K. & Wandless G. A. Calcareous nannofossil assemblage changes across the Paleocene–Eocene Thermal Maximum: Evidence from a shelf setting. *Mar. Micropaleontol.* **92**, 61-80 (2012).
3. Bralower T. J. & Self-Trail J. M. Nannoplankton malformation during the Paleocene-Eocene Thermal Maximum and its paleoecological and paleoceanographic significance. *Paleoceanography* **31**, 1423-1439 (2016).
4. Gibbs S. J., Bralower T. J., Bown P. R., Zachos J. C. & Bybell L. M. Shelf and open-ocean calcareous phytoplankton assemblages across the Paleocene-Eocene Thermal Maximum: Implications for global productivity gradients. *Geology* **34**, 233-236 (2006).
5. Bralower T. J. et al. Evidence for Shelf Acidification during the Onset of the Paleocene-Eocene Thermal Maximum. *Paleoceanogr. Paleoclimatol.* **33**, 1408-1426 (2018).
6. Shaw A. B. *Time in stratigraphy*. McGraw-Hill (1964).
