# Supplementary material for: Astrochronology of the Paleocene-Eocene Thermal Maximum on the Atlantic Coastal Plain
Source: Nat Commun. 2022 Sep 24;13:5618. doi: 10.1038/s41467-022-33390-x (PMC9509358; doi:10.1038/s41467-022-33390-x)
Supplement: Supplementary file 3 — Description of Additional Supplementary Files [file 41467_2022_33390_MOESM3_ESM.pdf]

Description of Additional Supplementary Files

File name: Supplementary Data 1

Description: Lithology information, biostratigraphic datums, the proxy series of calcium content, magnetic susceptibility, and carbon and oxygen isotopes.

File name: Supplementary Software 1

Description: Zipped cGENIE model files.
